# DRV concentrations and viral load in CSF in patients on DRV/r 600/100 or 800/100mg once daily plus two NRTI

**DOI:** 10.7448/IAS.17.4.19821

**Published:** 2014-11-02

**Authors:** Silvana Di Yacovo, Jose Molto, Elena Ferrer, Adrian Curran, Laura Jayne Else, Bonaventura Clotet, Juan Tiraboschi, Jordi Niubo, Antonia Vila, Daniel Podzamczer

**Affiliations:** 1 Infectious Diseases, Hospital Universitari de Bellvitge, Barcelona, Spain; 2 Fundacio LLuita contra la Sida, Hospital Germans Trias i Pujol, Badalona, Spain; 3 Infectious Diseases, Hospital Vall d'Hebron, Barcelona, Spain; 4 Bioanalytical Facility, Royal Liverpool University Hospital, Liverpool, UK; 5 Microbiology, Hospital Universitari de Bellvitge, Barcelona, Spain

## Abstract

**Introduction:**

Darunavir/r (DRV/r) is currently used at a dose of 800/100 mg once daily (OD) in a high proportion of patients. Pharmacokinetic data suggest that 600/100 OD may be effective, reducing toxicity and cost. However, drug concentrations in reservoirs such as cerebrospinal fluid (CSF) might not be adequate to inhibit viral replication. We aimed to evaluate concentrations of DRV and HIV-1 viral load (VL) in CSF patients receiving DRV 600/100 mg OD.

**Methods:**

DRV600 is an ongoing randomized open study comparing DRV/r 800/100 mg (DRV800) vs 600/100 mg (DRV600) OD plus TDF/FTC or ABC/3TC in 100 virologically suppressed patients (eudraCT 2011-006272-39). Here we present the results of a CSF sub-study. A lumbar puncture (LP) was performed in participating patients after at least six months of inclusion in the study, 20–28 hours after a dose of DRV/r. VL (PCR, LOD 40 copies/mL) was determined in CSF and in plasma. DRV concentrations were quantified in CSF by liquid chromatography mass spectrometry (LC/MS/MS) and in plasma using high-performance liquid chromatography (HPLC).

**Results:**

Sixteen patients were included (eight in each arm). All DRV600 patients and four out of eight DRV800 patients received TDF/FTC, and the other four ABC/3TC. 75% were males, median (range) age was 48 (17–71) years, CD4 cell count 532 cells/mL (190–1,394). Median total time on DRV/r was 30 (11–57) months, and since the beginning of the study 8 (6–12) months in DRV800 and 10 (7–12) months in DRV600 patients. LP was performed a median of 26 (24–28) hours after the last DRV/r+TVD or KVX dose. In DRV600 patients the median DRV plasma levels were 1,674 (326–3,742) ng/mL, CSF levels 17.08 (5.79–30.19) ng/mL and DRV CSF:plasma ratio 0.0084 (0.0028–0.0388), while in the DRV800 arm, median DRV plasma levels were 1,707 (958–3,910) ng/mL, CSF levels 13.23 (3.47–32.98) ng/mL and DRV CSF:plasma ratio 0.0104 (0.0018–0.0262). All patients had VL<40 copies/mL in plasma and 14 patients VL<40 copies/mL in CSF. Two patients (1 in each arm, and taking TDF/FTC) had detectable VL in CSF (280 and 159 c/mL). These patients had the lowest CSF DRV concentrations (5.47 and 3.47 ng/mL), with plasma DRV concentrations of 802 and 958 ng/mL respectively.

**Conclusions:**

CSF DRV concentrations and CSF VL were similar between patients receiving DRV/r 800/100 mg or 600/100 mg OD. Low CSF DRV concentrations might be associated with viral escape in CNS. This may be taken into account in patients receiving OD DRV/r. Larger studies should confirm these findings.

**Table 1:** CSF and plasma DRV concentrations and viral load according to treatment arm

N	Plasma DRV (ng/mL)	CSF DRV (ng/mL)	CSF: Plasma Ratio	Plasma VL (copies/mL)	CSF VL (copies/mL)	ARV Treatment

1	896	16.62	0.0120	<40	<40	600/100+TDF/FTC
2	802	5.79	0.0080	<40	280	600/100+TDF/FTC
3	326	7.14	0.0388	<40	<40	600/100+TDF/FTC
4	1390	23.04	0.0165	<40	<40	600/100+TDF/FTC
5	1957	10.56	0.0089	<40	<40	600/100+TDF/FTC
6	3235	30.19	0.0074	<40	<40	600/100+TDF/FTC
7	3742	17.54	0.0028	<40	<40	600/100+TDF/FTC
8	2363	23.99	0.0076	<40	<40	600/100+TDF/FTC
Median (DRV 600/100 mg)	1674	17.08	0.0084	<40	<40	
9	1890	11.42	0.0018	<40	<40	800/100+ABC/3TC
10	1569	13.81	0.0147	<40	<40	800/100+ABC/3TC
11	3910	32.98	0.0035	<40	<40	800/100+ABC/3TC
12	1258	17.96	0.0262	<40	<40	800/100+ABC/3TC
13	958	3.47	0.0173	<40	159	800/100+TDF/FTC
14	1876	12.02	0.0030	<40	<40	800/100+TDF/FTC
15	1845	12.65	0.0061	<40	<40	800/100+TDF/FTC
16	1389	23.12	0.0217	<40	<40	800/100+TDF/FTC
Median (DRV 800/100 mg)	1707	13.23	0.0104	<40	<40	

Abbreviations: VL: HIV-1 RNA viral load; CSF: Cerebrospinal fluid; DRV/r: Darunavir/r; ARV: Antiretroviral.

